# Effects of *Lutjanus erythropterus* Protein on Depression-like Behavior and Gut Microbiota in Stressed Juvenile Mice

**DOI:** 10.3390/foods14020330

**Published:** 2025-01-20

**Authors:** Jinjin Luo, Chen Wang, Weichang Ye, Ruiyang He, Ling Huang, Zhijia Fang, Qi Deng, Mei Qiu, Lijun Sun, Ravi Gooneratne

**Affiliations:** 1 College of Food Science and Technology, Guangdong Ocean University, Zhanjiang 524088, China; 2112203018@stu.gdou.edu.cn (J.L.); 18642326533@163.com (C.W.); yeweichang0_0@163.com (W.Y.); 202111221310@stu.gdou.edu.cn (R.H.); 2112203048@stu.gdou.edu.cn (L.H.); fzj4437549@163.com (Z.F.); gdoudengqi@163.com (Q.D.); suncamt@126.com (L.S.); 2 Department of Wine, Food and Molecular Biosciences, Faculty of Agriculture and Life Sciences, Lincoln University, P.O. Box 85084, Lincoln 7647, New Zealand; ravi.gooneratne2@lincolnuni.ac.nz

**Keywords:** adolescents, amino acid, depression, gut microbiota, *Lutjanus erythropterus* protein, short-chain fatty acids

## Abstract

*Lutjanus erythropterus* protein (Lep) exhibits anti-inflammatory effects, but its antidepressant activity is unknown. This study used a 44-day chronic unpredictable mild stress (CUMS) model to determine whether Lep has a beneficial effect through the gut–brain axis in 3-week-old male C57BL/6 mice. Gavaging with Lep solution alleviated the depression-like behavior and anxiety symptoms in CUMS growing mice. Administration of Lep decreased serum IL-1β, IL-2, IL-6, and TNF-α levels and restored colonic mucosal damage. In addition, Lep improved the disturbance of 5-hydroxytryptamine (5-HT) secretion in the gut–brain axis. Pearson analysis revealed that gut short-chain fatty acid (SCFAs) concentration significantly (*p* < 0.05) correlated with mucosal damage scores and the depression-like behavior index. Lep was able to prevent the gut SCFA enrichment. Lep upregulated gut *Muribaculaceae* and downregulated SCFA-producing bacteria by replenishing deficient amino acid (AA) (tryptophan, alanine, aspartate, glutamate) and decreased (*p* < 0.01) the gene abundance of the AA metabolism pathway of SCFA-producing bacteria, thereby preventing gut SCFA enrichment and alleviating associated depression-like behavior. These findings indicate that Lep could attenuate depression in CUMS juvenile mice via the gut microbiota-SCFA–brain axis.

## 1. Introduction

Depression is a chronic mental illness accompanied by symptoms that include poor mood and feeling unhappy, sad, dejected, and miserable, which can last for a few or several weeks [1,2,3]. The peak time in life for the onset of depression is the adolescence period [4]. Approximately 40% of children with depression experience recurrent episodes, 33% attempt suicide, and 3–4% commit suicide, thus making it a serious threat to adolescent life [5]. Nonetheless, the challenges of significant adverse effects and low efficacy of clinical first-line antidepressants such as selective serotonin reuptake inhibitors (SSRIs) in adolescents allow this condition to progress to an advanced stage [6,7]. Data indicate that up to 30% of teenagers who undergo treatment develop resistance [8]. Consequently, there is a pressing need to explore alternative strategies for preventing and controlling depression among adolescents.

Depression can be caused by multiple mechanisms, including lack of monoamine neurotransmitters, inflammation, oxidative stress, and gut–brain axis disorder (s) [9,10,11]. Among these, the most implicated are gut–brain axis disorders [12]. Alterations in gut microbiota composition and associated changes in short-chain fatty acids (SCFAs), a byproduct of microbiota, may lead to increased intestinal barrier permeability and the secretion of pro-inflammatory cytokines, which can trigger depression [13]. Acetic acid (HAc), propionic acid (PA), and butyric acid (BA) have antidepressant properties, including enhancing intestinal barrier integrity, regulating inflammatory signals and endocrine function [14,15]. However, these benefits occur only within the physiological concentration range. New investigations have indicated that increased HAc, PA, and isocaproic acid concentrations in the gut are linked to depression [2,16]. Increased BA concentrations lead to gut leakage, impairing the intestinal barrier and infiltration of cytokines into the brain via the circulatory system, thereby initiating depression [3]. This implies that reversing intestinal SCFA enrichment may help alleviate adolescent depression. Nevertheless, studies on reversing intestinal SCFA enrichment through dietary manipulation are scarce and need further investigation.

The onset of depression is closely associated with gut microbial amino acid (AA) metabolism disorders [17]. The gut microbiota and AA metabolism exhibit bi-directional regulation, with an imbalance in gut microbiota leading to an imbalance in AA metabolism resulting in anomalies in the SCFA and 5-hydroxytryptamine (5-HT) metabolite levels. Increased 5-HT levels in the brain alleviate depression [18]. In contrast, increased gut 5-HT levels lead to intestinal dysfunction and trigger a gut inflammatory response [19], resulting in gastrointestinal disorders such as colitis and irritable bowel syndrome [20]. Consequently, altered gut microbiota composition is expected to further regulate the AA metabolism and metabolites, alleviating adolescent depression [21].

Natural marine-derived fish protein products have been proven to alleviate depression symptoms [22,23], such as fish oil [24], fish protein hydrolysate [25], fish-derived peptides [26], and other substances rich in omega-3 polyunsaturated fatty acids (ω-3 PUFAs). In addition, clinical research has shown that fish hydrolysates can improve sleep disorders [25]. In vivo experiments have reported that fish protein hydrolysate plays a beneficial role in the body, such as anti-inflammatory, antioxidant, immune regulation, and neuroprotection [27]. Thus, protein originating from marine sources may have the potential to be developed as anti-depressant foods. In this regard, *Lutjanus erythropterus,* or red snapper, possesses a delicious flavor and contains abundant protein and ω-3 PUFAs [28] with significant antidepressant potential, but no studies have been reported.

Preliminary studies of our team revealed that Lep administration could reverse the increased serum inflammatory factors and the gene abundance of gut microbiota associated with AA metabolism caused by CUMS to improve depression-associated symptoms, confirming the antidepressant effects of Lep. In addition, Lep is abundant in AA and can modulate gut microbiota and its AA metabolism to sustain SCFA concentration. However, its mechanisms to alleviate adolescent depression remain unclear.

The objective of this study was to investigate the impact of Lep on depression-like behavior in CUMS mice and to investigate the potential anti-depression pathway associated with modulation of gut microbiota and AA metabolism by Lep, to provide theoretical evidence for the targeted regulation of SCFA levels to improve adolescent depression.

## 2. Materials and Methods

### 2.1. Lutjanus Erythropterus Protein Preparation

*Lutjanus erythropterus* was purchased from Huguang Market, Xiashan District, Zhanjiang City, Guangdong Province, China. Alkaline protease (≥200 U/mg) was from Shanghai Ruiyong Biotechnology Co., Ltd. Shanghai, China. It was based on the method of He et al. [29], with some modifications. The red snapper was placed on ice, and the head, skin, and internal organs were removed. Next, the red snapper was washed, cut into small pieces, and homogenized for 10 min with distilled water at 1:5 (*w*/*v*), and the pH of the solution was adjusted to 10 using 1 mol/L NaOH. Next, added alkaline protease (2400 U·g^−1^), was dissolved and left in a water bath at 45 °C for 1 h and 95 °C for 30 min to inactivate the enzyme. Following the chilling process, it was centrifuged (3577 rcf, 20 min) to remove the oil layer on the top and a semisolid layer at the bottom, ultimately obtaining the water-soluble protein solution. The water-soluble protein solution was freeze-dried by vacuum freeze-drying (LGJ-10) to obtain red snapper protein powder.

### 2.2. AA Determination

According to the methodology established by Khantaphant et al. [30], the automatic amino acid analyzer (Hitachi, L-8900, Beijing, China) was used to determine the AA composition of Lep. Referring to the method of Das et al. [31], the Kyller nitrogen analyzer (Gerhardt, Vapodest-450, Beijing, China) was used to determine the crude protein content of Lep.

### 2.3. Experimental Animal and Groups

Thirty-six healthy, pathogen-free (SPF grade) male C57BL/6 mice that were 3 weeks old and weighed an average of 10 ± 0.5 g were acquired from Tianqin Biotechnology Co., Ltd. in Changsha, China, with the batch number being SCXK [Beijing] 2016-0002. Animals were housed in a controlled temperature (22 ± 1 °C), and 12 h day/light cycle room with free access to food and water. After a week of adaptation, all mice were randomly divided into six groups (*n* = 6): control (CON), model (MOD), fish oil (FIS), CUMS + low-dose Lep (LSL), CUMS + medium-dose Lep (LSM), and CUMS + high-dose Lep (LSH). All animal tests and methodologies used in this study were approved by the Animal Ethics Committee of Guangdong Ocean University (approval number: GDOU-LAE-2020-005, 1 September 2020).

### 2.4. Chronic Unpredictable Mild Stress (CUMS) Model

The CUMS model used was based on published literature [3,11] with minor modifications. Specifically, eight mild stressors, including noise (92 dB, 1500 HZ) for 30 min, water deprivation or food deprivation for 24 h, continuous light for 24 h, slanted cage for 24 h, fasting for 24 h, cold water bath (12 °C) for 6 min, and tail suspension for 30 min, were applied to MOD, FIS, LSL, LSM, and LSH groups of mice for 44 d. Of these, two stressors were randomly presented for mice each day. No single stressor was applied on 2 consecutive days.

### 2.5. Lep Treatment of the Animal Model

The CUMS experiment lasted for 44 d. During 1–14 d, each group was gavaged with 5 mL/kg of distilled water. During 15–44 d, the fish oil groups were gavaged with fish oil (Wellness Pty Ltd., Melbourne, Australia) at 0.72 g/kg bw.d. The LSL, LSM, and LSH groups were gavaged with Lep at 0.45 g, 0.90 g, and 1.80 g/kg bw.d. The control and model groups were continued with 5 mL/kg of distilled water.

### 2.6. Behavioral Tests

Behavioral tests of the sucrose preference test (SPT), tail suspension experiment (TST), open field test experiment (OFT), and elevated plus maze experiment (EPM) were performed on all mice to assess the effects of Lep on CUMS mice. The behavioral tests were conducted on Day 45 and proceeded as previously described [11,32]. Briefly, SPT counted the weight of the bottles of 1% sucrose solution (*w*/*v*) before and after 24 h to calculate the rate of sugar water consumption. TST counted the last 4 min of immobility of mice out of a total of 6 min while they were suspended head down and their limbs could not touch the objects around them. OFT calculated the total time in the center area of the open space (40 cm × 40 cm × 40 cm) in the open field reaction box within a 6 min period. EPM calculated the time spent in the open arms composed of two open arms (30 cm × 6 cm) and two closed arms (30 cm × 6 cm × 15 cm) in 6 min.

### 2.7. Measurement of Serum Inflammatory Factors

After completing the behavioral tests, mice were anesthetized with sodium pentobarbital intraperitoneal injection and then euthanized by cervical dislocation. Next, blood was collected from the heart, centrifuged (3577 rcf, 20 min) at 4 °C, and the supernatant was collected. Serum tumor necrosis factor-α (TNF-α), interleukin-1β (IL-β), interleukin-2 (IL-2), and interleukin-6 (IL-6) levels were measured using an ELISA kit (Xinbosheng Bioscience and Technology Co., Ltd., Shenzhen, China). Serum levels of lipopolysaccharide (LPS) were quantified using an LPS horseshoe crab kit (Limulus Reagent Biotechnology Co., Ltd., Xiamen, China).

### 2.8. Colon Histopathology

After dissection, 2 cm of colonic tissue were quickly removed and intestinal contents were rinsed with physiological saline and immediately transferred into a 10% neutral formaldehyde fixative solution. After 24 h, the tissue was removed, trimmed, and embedded in paraffin wax, and 0.5 um sections were cut using a microtome and stained with hematoxylin/eosin (H&E) for light microscopy. We observe and score the colonic epithelial histopathological changes as shown in Table 1.

### 2.9. Determination of Fecal and Colonic Tissue SCFA Concentrations

Post-behavioral testing, 2 g of feces from each mouse were collected into a sterile 1.5 mL centrifuge tube and refrigerated at −80 °C. Colonic tissue samples were collected according to Part 2.8. After 50 mg of the sample were weighed, 100 μL of 15% phosphoric acid, 100 μL of isocaproic acid (125 μg/mL internal standard solution), and 900 μL of ether were added. The SRT-24 multi-sample quick grinding equipment was used to mill the mixture, and the supernatant was collected. The supernatant was filtered into a sample vial using a 0.22 μm membrane, and then a sample (20 μL) was injected into the gas chromatography–mass spectrometry (GC-MS) system for analysis. SCFA in mouse feces and colonic tissue was measured using gas GC-MS as described by Sun et al. [3].

### 2.10. Hippocampal, Colonic Tissue, and Fecal Neurotransmitters Measurement

At the time of dissection of mice, the hippocampal tissue was promptly collected on ice, subjected to rapid freezing in liquid nitrogen, and stored at −80 °C for later analyses. Liquid chromatography–mass spectrometry (LC-MS) was used to determine the 5-hydroxytryptamine (5-HT) concentrations in the hippocampus, colonic tissue, and feces by the method of He et al. [11]. A sample weighing precisely 50 mg was measured and subsequently combined with 2 mL of cold 0.2% formic acid in water. The intestinal homogenate was then prepared using an ultrasonic machine in an ice bath. Following this, 1 mL of the intestinal homogenate was transferred to 5 mL tubes containing 1 mL of ice-cold methanol (0.2% formic acid). The mixture was incubated at 4 °C for 30 min and then centrifuged at 4000 rpm for 20 min. The resulting supernatant was collected and filtered through a 0.22 μm organic filter membrane before being injected into the LC-MS system for analysis.

### 2.11. Gut Microbiota Analysis

On the last day of the test, mice’s feces were collected into 1.5 mL sterile centrifuge tubes, and the fecal samples were examined using 16S rDNA high-throughput sequencing. The sequencing of 16S rDNA amplicon and gut microbiota analysis was conducted by Hangzhou Guhe Co., Ltd., Hangzhou, Zhejiang Province, China. In summary, DNA was extracted using nucleic acid, quantified and assessed with a spectrophotometer, and analyzed via gel electrophoresis. The V4 region of the bacterial 16S rRNA gene was PCR-amplified with primers 515F and 806R. Post-amplification, PCR products were purified and quantified before sequencing on the Illumina NovaSeq platform. The strain data of the samples was acquired through comparison with the database, leading to the identification of the strain categories of the gut bacteria. The intestinal microbiota analyses were conducted as described by Sun et al. [3].

### 2.12. Statistical Analysis

One-way analysis of variance (ANOVA) was used in all statistical analyses, followed by least-significant-difference (LSD) comparison for post-hoc tests using SPSS (version 26.0). All data are presented as mean ± standard error of the mean (SEM). Plotting was performed using Origin 2024 software. Data correlation was analyzed using the Pearson method. *p* < 0.05 was considered statistically significant.

## 3. Results

### 3.1. Amino Acid Composition of Lep

See Table 2 for the amino acid composition.

### 3.2. Effect of Lep on Depression-like Behaviors of CUMS Mice

**Figure 1:**
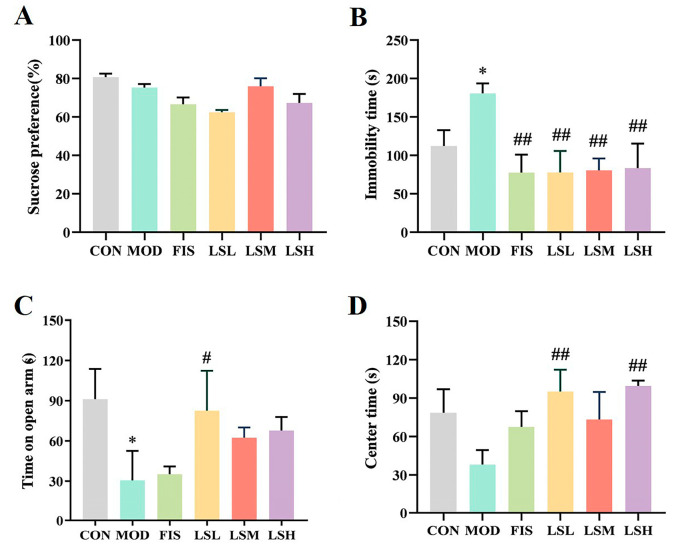
Ameliorative effect of *Lutjanus erythropterus* protein (Lep) on depression-like behavior. (**A**) Sucrose preference, (**B**) Immobility time, (**C**) Time in the central area, (**D**) Time in the open arms. Data are presented as the mean ± SEM (*n* = 6). Experimental groups are: CON, Control; MOD, Model; FIS, Fish oil; LSL, Lep low dose; LSM, Lep medium dose; LSH, Lep high dose. * *p* < 0.05, *p* < 0.01 vs. CON; ^#^ *p* < 0.05, ^##^ *p* < 0.01 vs. MOD.

As shown in Figure 1, the groups had no significant difference in sucrose preference. Compared with the control group, mice in the model group exhibited significantly increased (*p* < 0.05) immobility time and significantly decreased (*p* < 0.05) time in the open arm and the center region. This indicates that mice subjected to ongoing stress show behavior similar to depression. Following Lep administration, the immobility time was significantly reduced (*p* < 0.05), and the time spent in the open arm and central area was significantly increased (*p* < 0.05), demonstrating that Lep alleviated the depression-like behaviors of the mice.

### 3.3. Effects of Lep on Serum Inflammatory Factors

**Figure 2:**
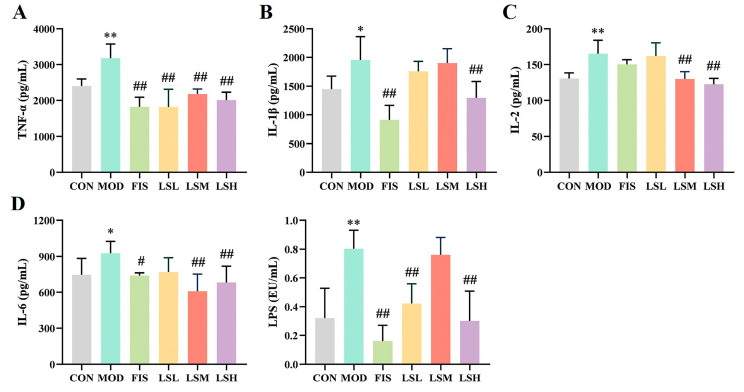
Ameliorative effects of *Lutjanus erythropterus* protein (Lep) on inflammatory factors. (**A**) TNF-α, (**B**) IL-1β, (**C**) IL-2, (**D**) IL-6. Data are presented as the mean ± SEM (*n* = 6). Experimental groups are: CON, Control; MOD, Model; FIS, Fish oil; LSL, Lep low dose; LSM, Lep medium dose; LSH, Lep high dose. * *p* < 0.05, ** *p* < 0.01 vs. CON; ^#^ *p* < 0.05, ^##^ *p* < 0.01 vs. MOD.

Compared to the control group, the serum levels of TNF-α, IL-1β, IL-2, IL-6, and LPS were significantly increased (*p* < 0.05) in the model group (Figure 2). In the administration of fish oil, the serum levels of TNF-α, IL-1β, and IL-6 were significantly decreased (*p* < 0.05) by 42.73%, 53.24%, and 20.20%, respectively, compared to the model group. In addition, Lep decreased the serum levels of inflammatory factors and LPS to different degrees, and the intervention effect was comparable to fish oil, suggesting that Lep inhibited the release of serum inflammatory factors and possessed anti-inflammatory effects.

### 3.4. Effects of Lep on Colonic Mucosa Damage

**Figure 3:**
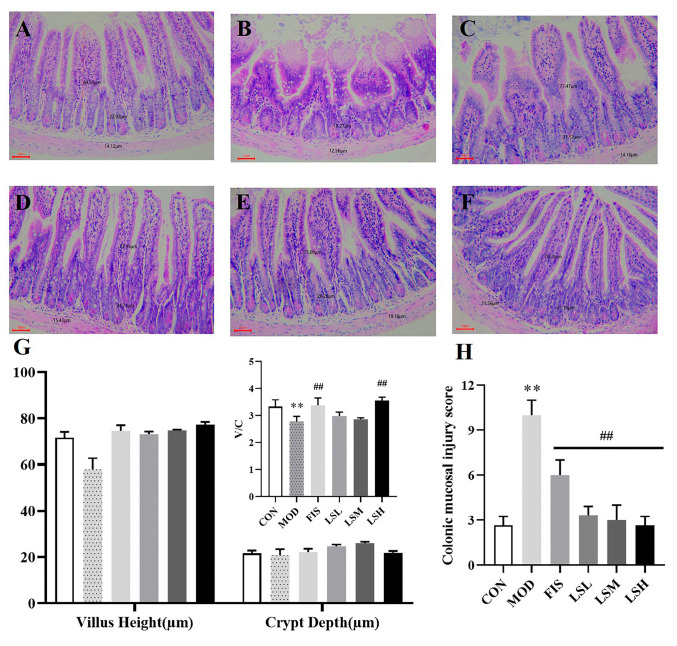
Effects of *Lutjanus erythropterus* protein (Lep) on colonic mucosal morphology. Experimental groups are: (**A**) Control, (**B**) Model, (**C**) Fish oil, (**D**) Lep low dose, (**E**) Lep medium dose, (**F**) Lep high dose, (**G**) Villus height (V), Crypt depth (C), and V/C ratio of HE-stained sections of colon tissue in each group, (**H**) Colonic mucosal damage scores. Data are presented as the mean ± SEM (*n* = 6). Experimental groups: CON, Control group; MOD, Model group; FIS, Fish oil group; LSL, Lep low-dose group; LSM, Lep medium-dose group; LSH, Lep high-dose group. *p* < 0.05, ** *p* < 0.01 vs. CON; *p* < 0.05, ^##^ *p* < 0.01 vs. MOD.

The control group displayed intestinal villi that were neatly aligned, with crypts maintaining normal morphology, and numerous goblet cells were found among the columnar cells of the mucosa (Figure 3). In the model group, intestinal tissue was severely damaged with rupture of the villi, shortening of the crypts, reduction of villi contact area, and decrease in goblet cells in the mucosal columnar cells gap. Villus height (V) and crypt depth (C) are major factors that indicate small intestinal growth and nutrient absorption and, hence, a reduction in the V/C ratio signifies mucosal injury and reduced nutrient absorption [33]. Compared to the control group, the model group showed a significant decrease in V/C values (*p* < 0.01) and a significant increase in the colonic mucosal damage score (*p* < 0.01). Following exposure to Lep, the intestinal villi and crypt morphology returned to normal, the number of goblet cells increased, and the villi and crypts were longer and intact, thus increasing the surface area, which would be more conducive for nutrient absorption. The V/C values of the fish oil and Lep groups also increased (*p* < 0.01), while the colonic mucosal damage scores decreased (*p* < 0.01) compared to the model group. These findings indicated that Lep alleviated the colonic mucosal damage.

### 3.5. Effects of Lep on Fecal and Colon Tissues SCFA Concentrations

**Figure 4:**
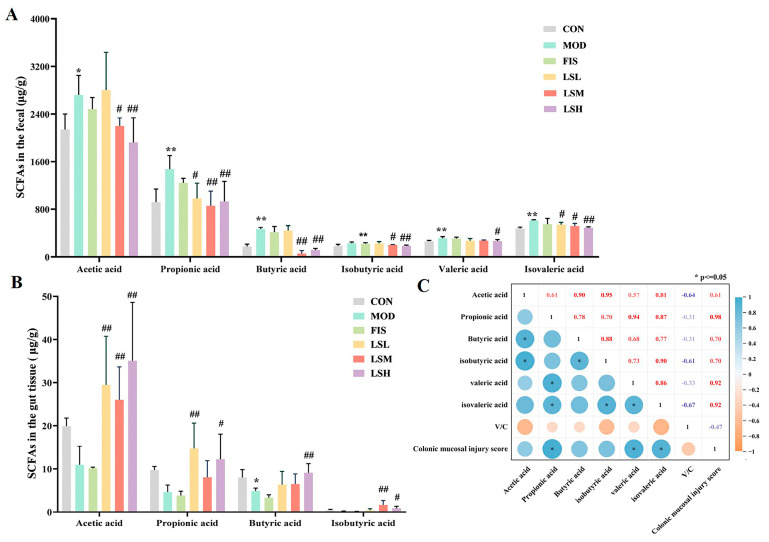
Feces and colon tissue short-chain fatty acid (SCFA) concentration in mice. (**A**) Fecal SCFA, (**B**) Colon tissue SCFA, (**C**) Correlation analysis between fecal and SCFA levels, villi height (V)/crypt depth (C) ratio, and colonic injury score. Data are presented as the mean ± SEM (*n* = 6). Experimental groups are: CON, Control; MOD, Model; FIS, Fish oil; LSL, Lep low dose; LSM, Lep medium dose; LSH, Lep high dose. * *p* < 0.05, ** *p* < 0.01 vs. CON; ^#^ *p* < 0.05, ^##^ *p* < 0.01 vs. MOD.

Fecal acetic acid (HAc), propionic acid (PA), butyric acid (BA), isobutyric acid (IBA), valeric acid (VA), and isovaleric acid (IVA) concentrations significantly increased (*p* < 0.05) in the model group compared to the controls (Figure 4A). Lep administration decreased the fecal SCFA concentrations in all Lep dosage groups correlating with a dose-dependent reduction in fecal concentrations of AA, IBA, VA, and IVA.

In model mice, the HAc, PA, BA, and IBA concentrations in the colon tissue decreased compared to the control group (Figure 4B). The BA concentrations were significantly lower (*p* < 0.05) than in the control group, and fish oil did not increase the BA concentration. However, following the administration of Lep, the colon tissue SCFA concentration increased significantly (*p* < 0.05), and this was most evident in the LSH. The fecal PA, VA, and IVA concentrations were also significantly (*p* < 0.05) positively correlated with colonic mucosal damage scores, while fecal AA, IBA, and IVA concentrations were negatively correlated with V/C (Figure 4C).

### 3.6. Effects of Lep on Hippocampi and Colon Tissue and Fecal 5-HT Concentrations

**Figure 5:**
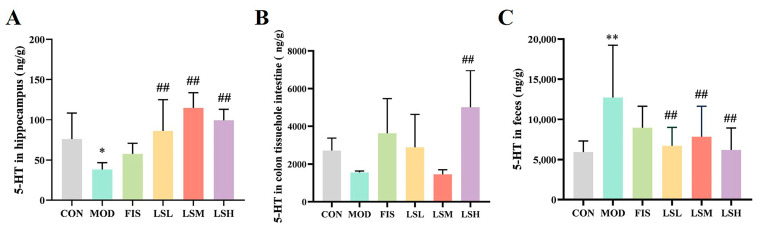
Ameliorative effect of *Lutjanus erythropterus* protein (Lep) on hippocampal and colonic tissue, and fecal 5-hydroxytryptamine (5-HT) concentrations. (**A**) Hippocampi, (**B**) Intestinal tissue, (**C**) Feces. Data are expressed as mean ± SEM (*n* = 6). Experimental groups are: CON, Control; MOD, Model; FIS, Fish oil; LSL, Lep low dose; LSM, Lep medium dose; LSH, Lep high dose. * *p* < 0.05, ** *p* < 0.01 vs. CON; *p* < 0.05, ^##^ *p* < 0.01 vs. MOD.

Reduced hippocampi 5-HT levels impair the brain’s emotional cognitive function, which can lead to depression. In the model group, the 5-HT levels in the mice hippocampi were significantly lower (*p* < 0.05) than in the control group (Figure 5C). In contrast, the 5-HT levels in the feces of the model group were significantly increased (*p* < 0.05) compared to the controls (Figure 5B). However, Lep treatment reversed the above changes. This indicates that prolonged stress can lead to metabolic changes in the serotonergic system in mice, and Lep alleviated this disorder.

### 3.7. Effects of Lep on Alpha and Beta Diversity of Gut Bacteria

**Figure 6:**
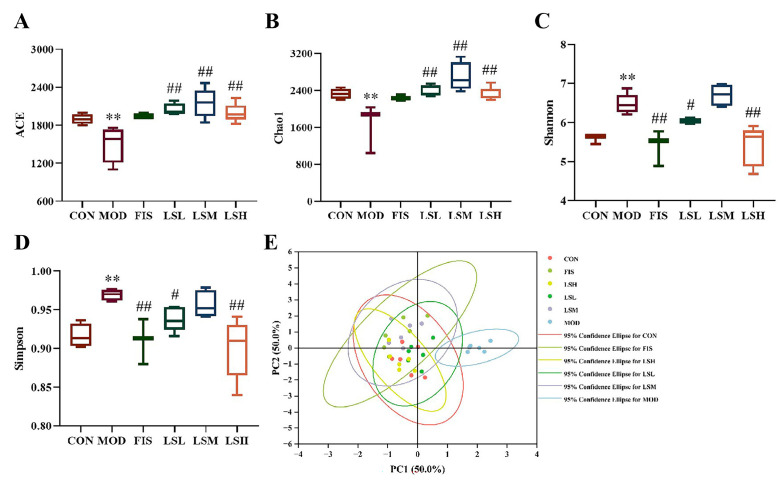
Effects of *Lutjanus erythropterus* protein (Lep) on alpha and beta diversities of gut microbiota. (**A**) Ace index, (**B**) Chao1 index, (**C**) Shannon index, (**D**) Simpson index, (**E**) PCA analysis of gut microbiota. Data are expressed as the mean ± SEM (*n* = 6). Experimental groups are: CON, Control; MOD, Model; FIS, Fish oil; LSL, Lep low dose; LSM, Lep medium dose; LSH, Lep high dose. *p* < 0.05, ** *p* < 0.01 vs. CON; ^#^ *p* < 0.05, ^##^ *p* < 0.01 vs. MOD.

In the model group, the Chao1 and Ace indices were significantly decreased (*p* < 0.01) compared to the control group. In contrast, the Shannon and Simpson indices were significantly increased (*p* < 0.01) (Figure 6). This indicates a decrease in richness and a marked alteration in the gut microbiota diversity. The confidence circles of the PCoA two-dimensional coordinate plot of the model group and the control group were well separated. On exposure to Lep, the alpha and beta parameters were markedly improved, with an increased richness of intestinal microbiota, decreased diversity of intestinal microbiota, and a greater overlap in the confidence ellipse of the control group. In general, the intervention effect of the LSH group was better than that LSL and LSM groups.

### 3.8. Effects of Lep on Gut Microbiota Species Composition

**Figure 7:**
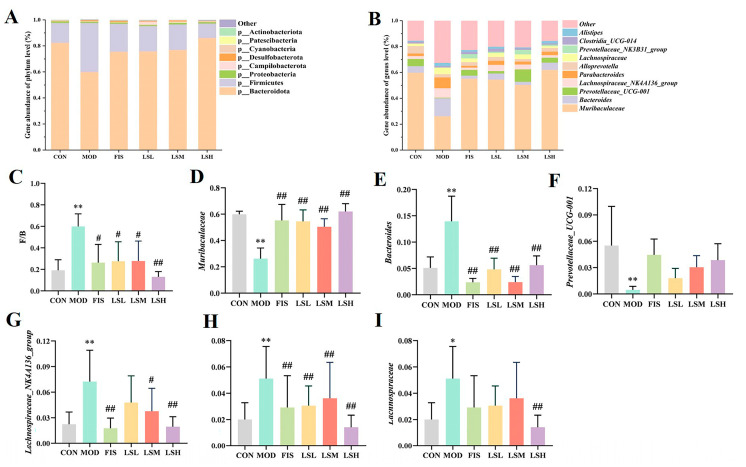
Effects of *Lutjanus erythropterus* protein (Lep) on genus level species composition of gut microbiota. (**A**) Histogram of species composition at the phylum level, (**B**) Histogram of species composition at the genus level, (**C**) Ratio of firmicutes and bacteroidetes between experimental groups, as well as gene abundance of: (**D**) *Muribaculaceae*. (**E**) *Bacteroides*. (**F**) *Prevotellaceae_UCG-001*. (**G**) *Lachnospiraceae_NK4A136_group*. (**H**) *Parabacteroides*. (**I**) *Lachnospiraceae*. Data are expressed as mean ± SEM (*n* = 6). Experimental groups are: CON, Control; MOD, Model; FIS, Fish oil; LSL, Lep low dose; LSM, Lep medium dose; LSH, Lep high dose. * *p* < 0.05, ** *p* < 0.01 vs. CON; ^#^ *p* < 0.05, ^##^ *p* < 0.01 vs. MOD.

The major phyla in the gut microbiota of mice were firmicutes (F) and bacteroidetes (B). The F/B value of the model group increased significantly (*p* < 0.01) compared with the control group. The F/B values of the fish oil and Lep dosage groups were lower (Figure 7C). This indicates that Lep was able to restore the changes to gut microbiota caused by CUMS. At the genus level, when the top 10 genera ranked in abundance at the genus level were selected for further analysis, it was clear that most genera were SCFA-producing bacteria, such as *Bacteroides* and *Parabacteroides* (belonging to the phylum Bacteroidetes), and *Lachnospiraceae_NK4A136_group* (belonging to the *Lachnospiraceae*). In the model group, the abundance of SCFA-producing bacteria, namely *Bacteroides*, *Lachnospiraceae_NK4A136_group*, *Parabacteroides*, *and Lachnospiraceae*, were significantly (*p* < 0.01) elevated, and the abundance of *Muribaculaceae* and *Prevotellaceae_UCG-001* declined significantly (*p* < 0.01) (Figure 7D–I). However, the administration of Lep significantly decreased (*p* < 0.01) the abundance of SCFA-producing bacteria and increased the abundance of *Muribaculaceae* and *Prevotellaceae_UCG-001*. These results suggest that Lep successfully restored the gut microbiota imbalance induced by CUMS.

### 3.9. Effects of Lep on Gene Abundance of Amino Acids Metabolic Pathways in the Gut Microbiota

**Figure 8:**
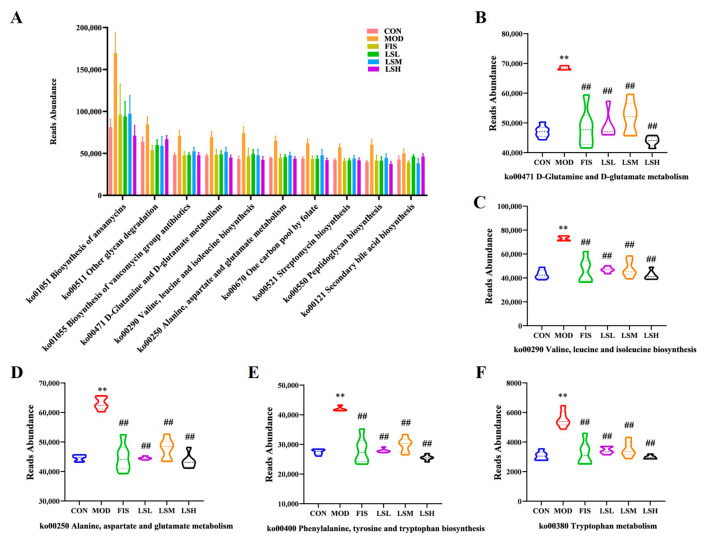
Prediction based on KEGG metabolic pathway and amino acid metabolism in gut microbiota. (**A**) KEGG-predictive pathways, (**B**) ko00401 D-glutamine and D-glutamate metabolism, (**C**) ko00290 valine, leucine, and isoleucine biosynthesis, (**D**) ko00250 alanine, aspartic acid, and glutamic acid biosynthesis, (**E**) ko00400 phenylalanine, tyrosine, and tryptophan biosynthesis, (**F**) ko00380 tryptophan metabolism. Data are expressed as mean ± SEM (*n* = 6). Experimental groups are: CON, Control; MOD, Model; FIS, Fish oil; LSL, Lep low dose; LSM, Lep medium dose; LSH, Lep high dose. *p* < 0.05, ** *p* < 0.01 vs. CON; *p* < 0.05, ^##^ *p* < 0.01 vs. MOD.

Based on the KEGG metabolic prediction database, the top 10 AA metabolic pathways (ko00401 D-glutamine and D-glutamate metabolism, ko00290 valine, leucine and isoleucine biosynthesis, ko00250 alanine, aspartate, and glutamate biosynthesis) and tryptophan metabolism (ko00400 phenylalanine, tyrosine and tryptophan biosynthesis, and ko00400 tryptophan metabolism) were analyzed. As shown in Figure 8, the gene abundance of AA metabolism pathways was significantly increased (*p* < 0.01) in the model group on exposure to CUMS. Following Lep intervention, the gene abundance of AA metabolism significantly decreased (*p* < 0.01), indicating that Lep could regulate the AA metabolism disorder in the gut microbiota of mice exposed to CUMS.

### 3.10. Correlation Analysis

**Figure 9:**
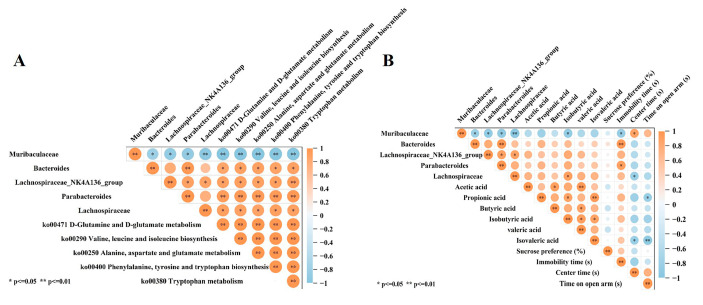
Correlation analysis. (**A**) Correlation analysis between the abundance of gut microbiota and amino acid metabolic pathways, and fecal 5-HT and SCFAs levels, (**B**) Correlation analysis between fecal SCFA concentrations, serum inflammatory factors, and behavioral indices. * *p* < 0.05, ** *p* < 0.01.

As shown in Figure 9, *Muribaculaceae*’s relative abundance showed a significant (*p* < 0.01) negative correlation with the gene abundance of AA metabolic pathways. The relative abundance of *Bacteroides*, *Lachnospiraceae_NK4A136_group*, *Parabacteroides*, and *Lachnospiraceae* showed a significant (*p* < 0.01) positive correlation with the gene abundance of AA metabolic pathways. In addition, gene abundance of *Muribaculaceae* relative abundance was negatively correlated with fecal IBA concentrations. The gene abundance of *Lachnospiraceae* was positively correlated with the fecal IBA concentrations. These results suggest that intestinal SCFA enrichment may be associated with disorders of gut microbiota AA metabolism.

As shown in Figure 9B, *Muribaculaceae*’s relative abundance showed a significant (*p* < 0.05) negative correlation with the immobility time in TST of mice and a significant (*p* < 0.05) positive correlation with the time mice entered the middle area. The relative abundance of *Bacteroides* and *Parabacteroides* showed a significant (*p* < 0.05) positive correlation with the immobility time in TST of mice. The relative abundance of *Lachnospiraceae* showed a significant (*p* < 0.05) negative correlation with the time mice entered the middle area. In addition, there was a significant (*p* < 0.05) negative correlation between fecal PA and IVA concentrations, and the time mice entered the open-arm region. Similarly, fecal IVA concentrations significantly (*p* < 0.05) and negatively correlated with the time mice entered the middle area. The above results indicate that gut SCFA enrichment, resulting from a disorder in intestinal microbial AA metabolism, may exacerbate depression-like behavior in mice.

## 4. Discussion

In adolescents, the hypothalamic–pituitary–adrenal (HPA) axis is susceptible to stressors, which can lead to depressive behavior [34]. Mice aged 3–4 weeks are frequently used in experiments to simulate physiological changes in human adolescence [32]. In this study, a 44-day CUMS juvenile mice model was applied to investigate the effects of *Lutjanus erythropterus* protein (Lep) on depression-like behavior. This study found that Lep significantly alleviated depression-like behavior evidenced by a reduction in immobility time and increased exploration time in unfamiliar environments of mice. In addition, Lep increased the time taken by mice to get into the open arm and central area of the maze much more effectively than with fish oil, indicating that Lep alleviated CUMS-induced depressive behavior. The antidepressant efficacy of high-dose Lep (1.80 g/kg mb·d) was the most effective. This study provides a theoretical foundation for developing Lep as a novel environmentally friendly and safe antidepressant therapy for adolescents. However, this study was conducted on a mouse model with a limited sample size. This may not sufficiently reflect human physiological and psychological responses and needs to validate these findings in larger animal models and human clinical trials.

The intestinal barrier is a multifaceted structure comprising luminal alkaline phosphatase, intestinal epithelial cells, a mucus layer, and antimicrobial peptides released by Paneth cells [35]. When the intestinal barrier is damaged, lipopolysaccharide (LPS), a harmful substance within the intestine, can readily infiltrate the bloodstream and bind to the CD14-Toll-like receptor-4 (TLR4) complex, thereby activating immune cells and signaling molecules. Such activation induces the release of pro-inflammatory factors, resulting in systemic inflammation and worsening intestinal mucosal damage [36]. This study’s results confirmed these earlier reports. Serum IL-6 and TNF-α levels are elevated in patients with depression, which can pass via the blood–brain barrier, triggering neuro-inflammation [37]. Lep exhibited the ability to reduce serum LPS, IL-6, and TNF-α levels in stressed mice and repair the colonic mucosal injury, confirming that Lep can modulate systemic and neuro-inflammation, thereby alleviating the depression-like behavior.

A reduction of gut SCFAs causes chronic stress, which can lead to depression, but increased SCFA levels can directly or indirectly alleviate depression-associated symptoms in animal models [38]. In contrast to the adult animal model depression, the present study observed that the gut SCFAs, namely HAc, PA, and BA concentrations, were significantly increased in adolescent mice and substantially linked with indications of depressive-like behaviour. Similar findings have been reported by Huang et al. [16]) and Li et al. [39], with persistent stress resulting in adolescent mouse brain and fecal concentrations of HAc, PA, BA, and VA increases. It was reported that increased BA concentrations can impede intestinal cell proliferation and development while enhancing intestinal permeability [40]. Elevated PA concentrations are associated with Alzheimer’s disease [41]. In addition, elevated fecal BA concentrations can induce damaged ileal tissue and exhibit a significant (*p* < 0.05) positive correlation with immobility time [3]. All these studies provide evidence that increased gut SCFA concentrations play a role in the onset of adolescent depression. Therefore, the ability of Lep to reverse gut SCFA enrichment could be a key mechanism behind its antidepressant benefits. Moreover, this study indicates that elevated concentrations of SCFAs require prime consideration in n future treatment protocols for adolescent depression.

Changes in SCFA, a metabolite of gut microbiota, are directly correlated with gut microbiota composition changes. Pearson correlation analysis showed a strong correlation between several of the top 10 gut microbiota and SCFAs. In particular, there was a positive correlation between *Lachnospiraceae* and isobutyric acid (IA) and a negative correlation between *Muribaculaceae* and IA. The relationship between four SCFA-producing bacteria (*Lachnospiraceae*, *Lachnospiraceae_NK4A136_group*, *Bacteroides, Parabacteroides*) and the gene abundance of AA metabolic pathways is significant with positive correlations, especially with fecal PA and IA concentrations. *Bacteroides* have a superior capacity to degrade nitrogenous compounds and synthesize SCFAs utilizing various AAs as nitrogen sources [42]. For example, *Lachnospiraceae* can ferment multiple gut substrates to produce HAC, PA, BA, and IBA [43]. Although it is clear that Lep modulates gut microbial composition to regulate SCFAs, the specific mechanism (s) by which Lep modulates the gut microbiota and its AA metabolism is still not known.

Reduced capacity to synthesize or release 5-HT in vivo is strongly associated with depression [44]. The microbial gut–brain axis is the primary neurotransmission pathway of 5-HT. The brains of those with depression exhibit diminished capacity for 5-HT release compared to healthy people [45]. Intestinal 5-HT can transmit information to the central nervous system via signal transduction and stimulate the synthesis of 5-HT in the brain [46]. However, this study found that increased intestinal 5-HT levels reduced hippocampal 5-HT levels. Li et al. [46] showed that intestinal 5-HT synthesis depletes substantial amounts of the precursor tryptophan. Probiotics can modulate serotonin metabolism, thereby diminishing intestinal 5-HT synthesis and facilitating the movement of increased tryptophan into the brain via the blood–brain barrier for 5-HT synthesis. In addition, the metabolic prediction of the gut microbiota showed a positive correlation between the gene abundance of the tryptophan metabolic pathways and the fecal 5-HT levels. Based on the results of the current study, we propose that Lep reduces the production of intestinal 5-HT by regulating gut microbiota and minimizing tryptophan metabolism, which results in an accumulation of intestinal tryptophan and an increase in 5-HT levels in the brain. However, the mechanism by which Lep regulates the gut–brain axis 5-HT requires further investigation to validate the above hypothesis.

Contrary to the previous reports of gut SCFAs and 5-HT enrichment, in the current study, the concentrations of SCFAs and 5-HT levels in colonic tissues significantly decreased (*p* < 0.05). This indicates that chronic stress impairs the absorption of SCFAs by intestinal tissues, resulting in damage to the intestinal barrier due to increased luminal SCFA concentrations. Fish oil had no intervening effect on this. Increased absorption of SCFAs from intestinal tissues and decreased 5-HT in intestinal tissues resulting from modified gut microbial composition have been reported to cause constipation and compromised intestinal barrier function [47,48]. However, Lep increased the 5-HT concentration of colonic tissue, potentially due to Lep enhancing the synthesis capability of intestinal tissue 5-HT synthase. Certain gut microorganisms are capable of producing 5-HT [49]. Whether this 5-HT in the gut can, in turn, regulate the growth of other gut microorganisms, thereby impacting the composition of microecology and the synthesis of metabolites, has attracted attention, which can be tested by in vitro experiments in the future.

Variations in gut microbiota composition that alter metabolic profiles are significant risk factors in disease progression [50]. The current study observed that alterations in gut microbiota corresponded with changes in the fecal metabolic phenotype in mice. Regarding *α* diversity, there was a marked increase in the diversity of mice gut microbiota, which is in contrast to the findings of Zhang et al. [10] in a partial animal model of depression, but in line with research that has demonstrated elevated fecal microbial diversity (based on the Shannon index) in patients with major depression [51]. This indicates that, in depression, the flora is characterized by an excessive rise or decrease in gut microbial diversity. An elevated firmicutes-to-bacteroidetes (F/B) ratio correlates with gut microbiota dysbiosis, higher energy use, and depression [10]. However, such abnormalities were significantly reversed by Lep, indicating that Lep was effectively able to modulate the CUMS-associated dysregulation of gut microbiota.

Changes in gut microbial composition (abundance and functionality) can cause some bacteria to assume a pivotal role. Members of the *Lachnospiraceae* family are recognized as significant SCFA producers, and their overabundance is indicative of a gut microbiota disorder that correlates with intestinal inflammation [39,52]. In addition, research indicates that *Bacteroides* stimulate peripheral cytokine production and increase inflammation [53], and that *Muribaculaceae* inhibits the colonization of harmful bacteria and enhances intestinal barrier function [54] and its abundance, which is widely reduced in patients with depression. This study demonstrated that Lep-treated mice displayed a unique gut microbiome that supports intestinal homeostasis, characterized by the downregulation of *Lachnospiraceae* and *Bacteroides*, as well as the upregulation of *Muribaculaceae*. Additionally, the variations of all the above bacteria abundance were markedly correlated with depression-like behavioral indicators, indicating that Lep may have reversed the gut SCFA enrichment by regulating the abundance of gut microbiota, thereby alleviating associated depression-like behavior.

Alterations in intestinal microbial composition affect the metabolic pathways and metabolite profiles of gut microbiota [55]. Individuals with major depressive disorder (MDD) demonstrate significant metabolic abnormalities in AA metabolism, specifically involving arginine, proline, phenylalanine, and tryptophan [53]. In UCMS rats exhibiting depression-like behavior, significant increases in arginine production, metabolic pathways involving arginine and proline, and concentrations of six amino acids (ornithine, cystathionine, phosphoethanolamine, beta-alanine, 4-hydroxyproline, and glutamic acid) were observed [56]. However, the depressive symptoms in the rats were greatly mitigated following the restoration of amino acid metabolism. The present study showed that Lep effectively reversed a substantial rise in the gene abundance of intestinal AA (glutamic acid, aspartic acid) metabolic pathways in growing mice, perhaps facilitating the alleviation of depressed symptoms induced by Lep. Regrettably, we did not detect the alterations in intestinal AA concentrations. Consequently, amino acid indicators that elicit depressed symptoms in CUMS pups could not be discerned.

Certain AAs can stimulate the growth of specific AA-dependent bacteria, while a lack or surplus of particular AA can inhibit the growth of AA-sensitive bacteria [57]. Furthermore, dietary AA, on reaching the gastrointestinal tract, is predominantly used by colonic microbes to generate metabolites, including ammonia and SCFAs [21]. Consequently, preserving the relative balance of the gut AA profile is important to sustain intestinal homeostasis. Macro-genomics forecasts that over 73% of the *Muribaculaceae* family can synthesize aspartate, glutamine, glutamate, glycine, methionine, valine, leucine, and isoleucine [58].

The present study revealed that the proportion of colonic *Muribaculaceae* in control mice was 59%, while in model mice exposed to chronic stress, it was significantly reduced to 26%, indicating that *Muribaculaceae* may be pivotal in the AA metabolism of gut microbiota. Under pre-chronic stress, the abundance of *Muribaculaceae* in the colon was progressively downregulated, potentially diminishing its AA metabolism and facilitating the growth of SCFA-producing bacteria that also utilize AA. With continuous chronic stress, the abundance of *Muribaculaceae* was significantly decreased, causing SCFA-producing bacteria to excessively ferment AAs for SCFA synthesis, which resulted in an accumulation of intestinal SCFAs and a depletion of AA. Lep treatment significantly reversed these changes as follows: during the pre-chronic stress phase, Lep provided the deficient AA (aspartate, glutamine, glutamine, alanine) in the intestine, thereby facilitating the growth of *Muribaculaceae*, whose abundance was progressively upregulated. Through sustained Lep intervention, *Muribaculaceae* fermented intestinal AA as nutrients for progressive growth, reinstating its dominance in the gut microbiota and inhibiting the growth of SCFA-producing bacteria that also utilize AA. These changes reversed the bacterial fermentation of AA to produce SCFAs, thus increasing gut SCFA concentrations and alleviating colon inflammation, resulting in alleviating depression-like behavior. Thus, gut microbiota and its metabolism play a key role and need further investigation to fully elucidate how Lep regulates the gut microbiota and its AA metabolism to affect gut SCFA concentrations.

## 5. Conclusions

In conclusion, CUMS-induced alterations in gut microbiota and AA metabolism, along with elevated gut concentrations of SCFAs, induced depression-like behavior in adolescent mice. Lep was able to maintain the balance of gut microbiota and AA metabolism by providing the depleted AA in the gut, reversing gut SCFAs and 5-HT enrichment. Lep also inhibited the release of serum inflammatory factors and ameliorated gut–brain 5-HT dysfunction by regulating gut microbiota. Thus, Lep facilitated the regulation of the microbiota–gut–brain axes to alleviate depression-like behavior. These findings show that gut SCFA levels could be used to identify adolescent depression, and modulating gut SCFA levels using Lep could be used to treat adolescent depression.

## Figures and Tables

**Table 1:** Colonic mucosal injury scale.

	Evaluation Indicator	
	Degree of Inflammation Damage	Degree of Crypt Damage
Evaluation scale (points)	None (0 points) Mild (1 points) Moderate (2 points) Severe (3 points)	None (0 points) 1/3 damage of crypts (1 points) 2/3 damage of crypts (2 points) Loss of crypts, retention of epithelium (3 points) Loss of crypts and epithelium (4 points)
	Degree of inflammation infiltration	Demage proportional composition
Evaluation scale (points)	None (0 points) Mucous membrane layer (1 points) Submucosa (2 points) Entire mucous membrane (3 points)	0 damage (0 points) 1–25% damage (1 points) 26–50% damage (2 points) 51–75% damage (3 points) 75–100% damage (4 points)

**Table 2:** Amino acid composition of *Lutjanus erythropterus* protein (Lep).

Amino Acid	Percentage/%	Amino Acid	Percentage/%
Aspartate (Asp)	5.88%	Isoleucine (Ile)	2.53%
Threonine (Thr)	2.71%	Leucine (Leu)	4.59%
Serine (Ser)	2.38%	Tyrosine (Tyr)	1.59%
Glutamate (Glu)	9.09%	Phenylalanine (Phe)	2.14%
Glycine (Gly)	3.21%	Lysine (Lys)	5.51%
Alanine (Ala)	3.75%	Proline (Pro)	1.19%
Cysteine (Cys)	0.29%	Histidine (His)	1.37%
Valine (Val)	2.89%	Arginine (Arg)	3.47%
Methionine (Met)	0.84%	Other	21.15%
Crude protein content/%		74.56%	

## Data Availability

The original contributions presented in the study are included in the article, further inquiries can be directed to the corresponding author.
